# Thalamic functional dysconnectivity in patients with left-hemisphere chronic capsular and pontine stroke

**DOI:** 10.3389/fnins.2024.1451307

**Published:** 2024-10-15

**Authors:** Jun Guo, Hongchuan Zhang, Jingchun Liu, Caihong Wang, Chen Cao, Jingliang Cheng, Chunshui Yu, Wen Qin

**Affiliations:** ^1^Department of Radiology, Tianjin Huanhu Hospital and Tianjin University HuanHu Hospital, Tianjin, China; ^2^Department of Radiology, Tianjin Key Lab of Functional Imaging and Tianjin Institute of Radiology, Tianjin Medical University General Hospital, Tianjin, China; ^3^Department of Radiology, Yijishan Hospital of Wannan Medical College, Wuhu, China; ^4^Department of Magnetic Resonance Imaging, The First Affiliated Hospital of Zhengzhou University, Zhengzhou, China

**Keywords:** pontine stroke, capsular stroke, thalamus, functional connectivity, subfields

## Abstract

**Background:**

Through its extensive connection with the cortex, the thalamus constitutes the hub of cortico-subcortical circuits and participants in multi-dimensional functions. However, the differential involvements of thalamic functional connectivity in chronic capsular and pontine stroke are still unknown.

**Methods:**

The research recruited 66 left-lesion chronic stroke patients, including 46 capsular strokes (CS) and 20 pontine stroke (PS) patients, and 67 normal controls (NC). The thalamic subfields functional connectivities were compared between groups using a two-way repeated analysis of variance (ANOVA), corrected for confounders including age, gender, education and scanners. Spearman partial correlation was used to explore the potential association between altered thalamic FC and clinical variables.

**Results:**

The ipsilesional thalamus of CS patients had abnormally decreased FC with widespread cognitive-related areas while increased FC with visual- and somatic-motor areas. In contrast, the ipsilesional thalamus of PS patients mainly demonstrated increased FC in these sensorimotor areas. Even in the contralesional thalamus, we observed similar (with the ipsilesional) but less extensive functional dysconnectivity patterns in both the CS and PS patients (*P* < 0.05, corrected using family-wise error [FWE] at the voxel level). Finally, we found significant group x subfields interactions on thalamic functional connectivity, where capsular vs. pontine stroke demonstrate varied functional dysconnectivity with specific thalamic subfields. Finally, a weak correlation was found between FC of both ipsilesional/contralesional thalamic subfields and motor, working and verbal memory.

**Conclusions:**

The thalamic functional dysconnectivity after chronic stroke are lesion-location and subfields dependent. Moreover, functional dysconnectivity were shown in both the ipsilesional and contralesional thalamus with similar patterns.

## Introduction

The thalamus is traditionally considered the brain’s central sensory and motor transmission station (Fama and Sullivan, 2015). Moreover, through its extensive connections with the frontoparietal cortices, the thalamus constitutes the hub of cortico-subcortical circuits related to multi-dimensional cognitive functions (d’Ambrosio et al., 2017; Goldstone et al., 2018), including attention, memory, execution, and consciousness controlling (Fama and Sullivan, 2015; Stebbins et al., 2008). Ischemic stroke could damage thalamus nuclei and their connections, which further leads to a range of neurological disorders, including sensory loss, motor impairments, and cognitive decline (Fernandez-Andujar et al., 2014). However, the thalamic functional connectivity disruption patterns caused by stroke and their relationship with functional impairment have not been fully elucidated.

Previous evidence indicates stroke in different regions could cause dramatic heterogeneity in brain structure and functional impairment and remodeling, such as local brain morphological (Jiang et al., 2017; Liu et al., 2019; Stebbins et al., 2008); local activity (Crofts et al., 2019; Obayashi, 2019; Ward et al., 2003), and anatomical connectivity changes (Wang et al., 2019; Yan et al., 2022). These differences may be a primary reason for the diverse clinical symptoms and rehabilitation capacities among stroke patients (Cao et al., 2021; Carrera and Bogousslavsky, 2006). However, prior research has often focused on the average effect on thalamic damage, neglecting the specific impact of strokes at different lesion locations on thalamic functional connectivity (Aswendt et al., 2020; Boychuk et al., 2015; Ilves et al., 2022). Previous studies demonstrated that CS and PS can result in distinct patterns of structural damage and reorganization within the cerebral cortex, with these variations serving as a foundation for tailoring personalized rehabilitation strategies for individual patients (Guo et al., 2019; Jiang et al., 2017). Thus, this study focused on the distinct disruption patterns of thalamic functional connectivity caused by pontine strokes and capsular strokes. The pons is a crucial pathway connecting the cerebral cortex and descending structures such as the cerebellum and spine, while the internal capsule contains several important association and projections fibers connections the cortex, thalamus, ganglia nuclei and descending structures (Boychuk et al., 2015; Wang et al., 2018). Their neuroanatomical differences suggest potential variations in functional connectivity disruption post-stroke: pontine strokes may disrupt information transmission between the descending structures and cortical regions, whereas capsular strokes may directly affect communication efficiency within the cortex, and between cortical and subcortical structures. Differences in stroke patients’ prognosis may be attributed to variations in the lesion location-dependent functional connection of the thalamus. By comparing the differences and similarities in thalamic functional connectivity disruptions between these two stroke types, we can gain a deeper understanding of the specific processes and mechanisms of neural network remodeling after stroke, which can also offer valuable insights for the development of personalized rehabilitation strategies.

Additionally, the thalamus has traditionally been considered as a single structure in most neuroimaging studies (Herve et al., 2005; Liu et al., 2019). However, recent research has revealed significant heterogeneity within the thalamus, with different subfields showing considerable anatomical and functional differences. Given the varying degrees of density within its interior nuclei, the thalamus, positioned centrally as a significant collection of gray matter, becomes particularly susceptible to harm in the event of head trauma (Zhou, 2017). In prior research endeavors, diverse methodologies have been employed for segmenting thalamic subfields. For example, the probabilistic tractography algorithm grounded on diffusion MRI data is often used to segment thalamic subfields (Behrens et al., 2003). Each thalamic subfield has distinct connectivity patterns and functional roles with the cerebral cortex and other brain areas (Beloozerova and Marlinski, 2020; Hwang et al., 2017; Lambert et al., 2017; Li et al., 2023). For instance, the pulvinar is highly connected with the visual cortex and is associated with visual perception and attention; the mediodorsal nucleus is primarily connected with the prefrontal cortex and cingulate, involved in emotion and memory processing; and the ventral posterolateral nucleus is mainly connected with the sensory cortex, related to sensory information transmission and integration. It is worth noting that prior research on post-stroke thalamic functional connectivity has often treated the thalamus as a whole (He et al., 2020; Ilves et al., 2022), rarely addressing whether specific subfields are selectively affected. Moreover, little research has shown whether the infarct location influences the involvement of thalamic subfields.

Therefore, this study aims to investigate the thalamic subfields’ functional dysconnectivity patterns caused by two types of strokes—pontine versus capsular strokes. Specifically, we focused on (1) the similarities and differences in thalamic functional dysconnectivity in patients with pontine versus capsular stroke, and (2) whether there were significant differences in the involvement of thalamic subfields between the two types of stroke patients. This study will not only reveal the unique mechanisms of thalamic neural functional changes due to pontine and internal capsule infarctions but also potentially help provide a more specific subfield-level target for clinical intervention strategies.

## Materials and methods

### Subjects

In the current study, a total of 66 patients with chronic left-lesion ischemic stroke were enrolled, among whom 46 presented with capsular stroke and 20 with pontine stroke. Additionally, 67 healthy subjects were recruited as normal controls (NC) for comparison purposes. All of these subjects came from three hospitals. All enrolled patients must meet the following criteria: Firstly, it must be a first-time ischemic stroke and exhibited motor dysfunction at stroke onset; Secondly, the lesion, which was singular, involved the left internal capsule or pons; Furthermore, the time interval between stroke onset and enrollment should exceed six months; In addition, right-handedness before the stroke. Some exclusion criteria need to be followed: (1) a history of recurrent stroke as confirmed by clinical history or imaging examination; (2) the presence of any other intracranial abnormalities (e. g., tumors, vascular malformations); (3) a history of psychiatric disorders or drug dependence; (4) left-handedness before the stroke; and (5) insufficient image quality. Participant demographics are detailed in Table 1.

**TABLE 1:** Demographic and clinical information pertaining to patients with stroke and normal controls.

	Capsular stroke	Pontine stroke	Normal controls	Statistics	*p*-value
Age (year)	43–75 (55.15 ± 8.01)	49–69 (58.55 ± 6.29)	40–75 (56.43 ± 7.12)	*F* = 1.518	0.223
Gender (M/F)	36/10	13/7	46/21	χ*2* = 1.709	0.425
Education (years)	9.78 ± 3.23	9.15 ± 2.83	10.47 ± 2.22	*F* = 1.759	0.176
FMA	91.76 ± 19.26	89.55 ± 23.98	/	*/*	*/*
RAVLT-SR	42.09 ± 9.61	38.55 ± 7.78	/	*/*	*/*
RAVLT-LR	9.89 ± 3.27	9.45 ± 2.69	/	*/*	*/*
N_ACC	0.90 ± 0.15	0.86 ± 0.15	/	*/*	*/*
N_RT	899.12 ± 293.82	943.48 ± 185.09	/	*/*	*/*
S_ACC	0.88 ± 0.11	0.86 ± 0.18	/	*/*	*/*
S_RT	959.90 ± 279.20	942.70 ± 223.93	/	*/*	*/*
Timing of follow-up imaging (days)	627.04 ± 410.24	488.74 ± 258.57	/	*t* = 1.359	0.179
Lesion volume at chronic stage (ml)	216.57 ± 38.13	67.8 ± 13.85	/	*t* = 3.667	0.001

CS, Capsular stroke; PS, Pontine stroke; NC, normal control; M, Male; F, Female; RAVLT_SR, Rey Auditory Verbal Learning Test short-term; RAVLT_LR, Rey Auditory Verbal Learning Test long-term; FMA, Fugl-Meyer Assessment; N_ACC, Number 1-back accuracy; N_RT, Number 1-back reaction time; S_ACC, Spatial 1-back accuracy; S_RT, Spatial 1-back reaction time.

The ethics committee of the local medical institutions approved the experimental protocol, and written informed consent was obtained from all participating individuals prior to their inclusion in the study.

### Neuropsychological assessment

All patients underwent a comprehensive neuropsychological assessment, commonly employed for evaluating recognition function in clinical settings. The Rey Auditory Verbal Learning Test (RAVLT) was utilized to measure verbal learning and memory, encompassing both short-term and long-term memory (Hochstenbach et al., 1998). The working memory (WM) test comprised a number 1-back and a spatial 1-back. Eprime 2 software was employed to capture the accuracy (ACC) and reaction time (RT) of the participants. The 1-back task is a continuous processing model widely used in neuroscience research for effectively assessing WM state. The Fugl-Meyer Assessment (FMA) was employed to evaluate the motor capabilities of patients.

### Magnetic resonance imaging data acquisition

Magnetic Resonance Imaging (MRI) data were acquired using three 3.0T MR scanners from three hospitals. Among them were two Discovery MR750 scanners from GE Healthcare and one Trio Tim MR scanner manufactured by Siemens. For acquiring 3D T1-weighted images on the MR750 scanners, the brain volume (BRAVO) sequence was utilized with the specified parameters outlined below: repetition time (TR) = 8.14 ms, echo time (TE) = 3.17 ms, inversion time (TI) = 450 ms, matrix = 256 × 256, flip angle = 12°, field of view (FOV) = 256 mm × 256 mm, voxel size = 1mm × 1mm × 1mm, thickness = 1mm, slices = 188; Utilizing the Trio Tim scanner, 3D T1-weighted (T1W) images were acquired through the magnetization prepared rapid acquisition gradient echo (MPRAGE) sequence. The specific imaging parameters employed were: TR = 2000ms, TE = 2.26ms, TI = 900ms, FOV = 256mm × 232mm, flip angle = 9°, matrix = 256 × 232, slices = 192, and thickness = 1mm, resulting in a 1-mm isotropic voxel.

The resting-state functional MRI (rsfMRI) data were gathered by utilizing the gradient-echo single-shot echo-planar imaging sequence, which employed specific imaging parameters for different scanners. For the MR750 scanner, FOV = 240 mm × 240 mm, slices = 38. For the Trio Tim scanner, FOV = 220 mm × 220 mm, slices = 36. All scans were conducted using identical parameters, including TR = 2000 ms, TE = 30ms, matrix = 64 × 64, slice thickness = 3 mm, flip angle = 90°, gap = 1mm, and volumes = 180.

### fMRI data preprocessing

The rsfMRI data were subjected to preprocessing utilizing the specialized Data Processing Assistant, tailored for the processing of rsfMRI (DAPBI 2.3),^1^ which is based on SPM12.^2^ The first 10 volumes were deleted to avoid scanner instability. The remaining 170 volumes were corrected for different slice timing across slices. The head motion was estimated and corrected using rigid realignment, and subjects with head motion displacement exceeding 2 mm or rotation exceeding 2° were excluded. During the spatial normalization steps, the structural images of subjects were coregistered to the fMRI using linear transformation. The structural images were segmented into tissue components and normalized to Montreal Neurological Institute (MNI) space using the Diffeomorphic Anatomic Registration Technique based on Exponentiated Lie algebra (DARTEL) algorithm. The deformation parameters from the DARTEL algorithm are then used to write the functional magnetic resonance imaging (fMRI) data into MNI space and resliced to 2mm isotropic voxels. Then, the fMRI data underwent regression analysis to eliminate several nuisance covariates, encompassing the Friston 24 head motion parameters, white matter, ventricle, and volumes with Power frame-wise displacement (FD) > 0.5. For the calculation of FD, we used the method proposed by Power et al. (Power et al., 2012). Specifically, FD is measured by an empirical scalar to express the instantaneous (frame-wise) head movement between neighboring time points: FD = |Δdix| +|Δdiy| +|Δdiz| +|Δαi| +|Δβi| +|Δγi|, in which Δdi = di–d (i−1), representing the displacement between the current time point i and the previous one. Additionally, we converted the rotational displacement in degrees into millimeters by calculating the displacement on the surface of a sphere with a radius of 50 millimeters, approximately corresponding to the average distance from the cerebral cortex to the center of the head. After calculating the FD, we identified the head motion time points with FD > 0.5 mm and treated them as random-effect covariates, incorporating them as additional regressors at the nuisance regression step. Then bandpass filtering (0.01–0.10HZ) was further applied to remove non-neural signals. Finally, the functional images were smoothed using a Gaussian kernel with full-width half-maximum of isotropic 6-mm.

### Functional connectivity calculation

Brainnetome Atlas (BNA)^3^ was used to define the thalamic subfields, including medial prefrontal thalamus (mPFtha), premotor thalamus (mPMtha), sensory thalamus (Stha), rostral temporal thalamus (rTtha), posterior parietal thalamus (PPtha), occipital thalamus (Otha), caudal temporal thalamus (cTtha), and lateral prefrontal thalamus (lPFtha) for each side. BNA was generated by dMRI data and probabilistic fiber tractography to derive a connectivity architecture, which segements the brain into 246 cortical and subcortical regions, with each thalamus divided into 8 subfields (Fan et al., 2016). BNA offers several updates compared to the thalamic atlas created by Behrens et al. (FSL atlas) (Behrens et al., 2003), despite both atlases relying on dMRI-based tractography. Specifically, the BNA employs a full data-driven spectral clustering approach, which is grounded on the cross-correlation matrix of structural connectivity patterns among all thalamic voxels, while FSL atlas adopts a “winner-take-all” strategy to parcel the thalamic voxels with the highest connection probability with 7 pre-defined cortical regions; Secondly, BNA used the HCP dMRI data with both higher spatial and angular resolutions than those for FSL atlas, and construct multiple cross-fibers within a single voxel rather than only one main fiber for FSL atlas. The average fMRI signals for each thalamic subfield were extracted from the preprocessed fMRI data but without smoothness. Then, a seed-based functional connectivity (FC) map was generated between the temporal sequences of each thalamic subfield and each brain voxel from the full preprocessed fMRI data using Pearson correlation and Fisher r-to-z transformation, resulting 8 FC maps for each hemisphere of each subject.

### Probability maps of stroke lesions calculation

**FIGURE 1:**
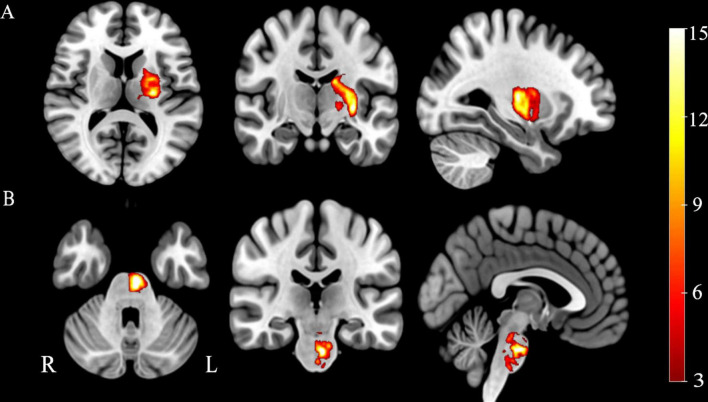
Probability maps of stroke lesions. **(A)** Capsular stroke, **(B)** Pontine stroke. Color bar represents the lesion probability across patients.

Firstly, the location of every stroke patient’s lesion was meticulously pinpointed by an accomplished neuroradiologist utilizing the MRIcron software. The lesions were manually delineated on the native T1-weighted images, and an individualized lesion mask was yielded for each patient. Secondly, these T1-weighted images underwent spatial normalization to align them with the MNI standard space. Then, we used the registration parameters to coregister the lesion masks to the standard space. Ultimately, we aggregate all the lesion masks in the standard space and then divide by the number of patients to obtain a group-wise probability map of the stroke lesions. The lesions’ probability maps are shown in Figure 1.

### Statistical analysis

For each hemisphere, we used a flexible general linear model (GLM) to construct a two-way repeated analysis of covariance (ANCOVA) to compare the thalamic voxel-wise FC differences among the CS, PS, and NC, with age, gender, education, and scanners were considered nuisance covariates. Specifically, in the flexible GLM, we defined groups (CS, PS, NC) and thalamic subfields (8 subregions) as fixed effects and subjects as random effects. It should be noted that this model can simultaneously test the global inter-group FC difference common to all subfields (main effect of groups) and test the subfield-specific inter-group FC difference (groups × subfields interaction). This unified model (considering all subfields together into one flexible GLM) can dramatically reduce the type I error caused by multiple comparisons compared to separately comparing the differences in each subfield one by one. Moreover, it allows a direct comparison of whether the inter-group FC differences across different subfields are diverse (interaction effect). A family-wise error (FWE) method was performed to correct for multiple comparisons (*P* < 0.05). ROI-based Spearman partial correlation analyses (controlling for the effects of age, gender, education, and scanners) were performed to test the associations between the abnormal FC of the thalamus and clinical variables in the patients with stroke (*P* < 0.01).

## Results

### Main effects of inter-group differences in thalamic functional connectivity

**FIGURE 2:**
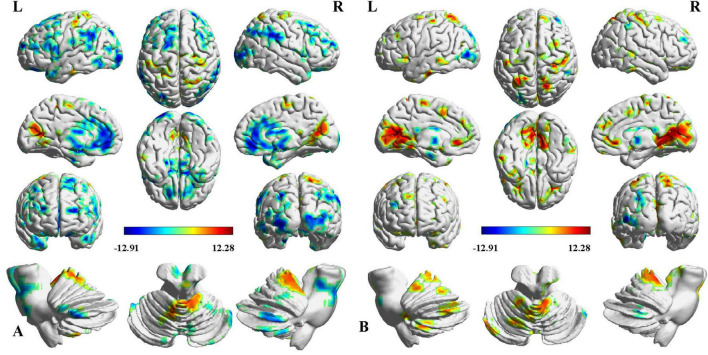
Intergroup differences in ipsilesional thalamic functional connectivity (FC) after stroke. **(A)** CS vs. NC, **(B)** PS vs. NC. Red indicates increased FC, and blue denotes decreased FC (*P* < 0.05, voxel-wise FWE correction). Color map represents the *T*-values.

**FIGURE 3:**
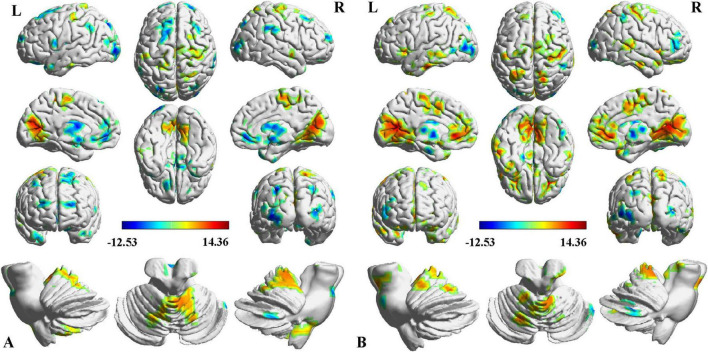
Intergroup differences in contralesional thalamic functional connectivity after stroke. **(A)** CS vs. NC, **(B)** PS vs. NC. Red indicates increased FC, and blue denotes decreased FC (*P* < 0.05, voxel-wise FWE correction). Color map represents the *T*-values.

Two-way ANCOVA identified significant intergroup differences in overall thalamic functional dysconnectivity irrespective of subfields (*P* < 0.05, voxel-wise FWE correction) (Figures 2, 3 and Table 2). Specifically, compared with the NC, the ipsilesional thalamus of CS patients had decreased FC with widespread cognitive-related regions such as bilateral medial prefrontal gyrus (MPFG), supramarginal gyrus (SMG), orbitofrontal cortex (OFC), temporal pole, and left inferior frontal gyrus (IFG), while demonstrated significantly increased FC with sensorimotor areas such as bilateral calcarine (CAL), postcentral (PoCG) and precentral gyrus (PreCG), and cerebellar lobe-6 (CB-6) (Figure 2A). In contrast, PS patients predominantly demonstrated more widespread increased thalamic FC with the bilateral sensorimotor areas (visual cortex, PoCG, PreCG, cerebellar lobes), and ventral lateral frontal areas such as the inferior frontal gyrus (IFG) and OFC, and only showed decreased FC with contralesional caudate and Cerebellum_Crus1 (Figure 2B). Moreover, the contralesional thalamus of CS patients showed decreased FC with bilateral MPFC, left middle occipital gyrus (MOG), superior frontal gyrus (SFG), bilateral temporal pole, caudate and left OFC, while increased FC with bilateral visual, PoCG, PreCG and cerebellum (Figure 3A). Finally, PS patients mainly demonstrated increased thalamic FC with the sensorimotor cortices, and decreased FC with the contralesional caudate that is very similar to the ipsilesional one (Figure 3B). To clarify whether the changed FCs between the thalamus and other regions are positive or negative, we additionally extracted the FCs of the peak regions with inter-group differences and carried out ROI-based comparisons. As shown in Supplementary Figures 1, 2, we found that the mean FCs between thalamus and all the identified ROIs of CS, PS and NC groups were positive.

**TABLE 2:** Regions showing functional dysconnectivity in chronic stroke patients.

Seeds	Targets	Peak MNI coordinate			Peak T value	Cluster size (voxels)
**Ipsilesional Thalamus**		**x**	**y**	**z**		
**CS vs. NC**						
L_CAL		[−15	−66	9]	12.281	3807
R_SMG		[−27	−6	−12]	−12.915	8825
R_MOG		[21	−102	6]	−10.504	470
L_MOG		[−27	−99	−9]	−10.980	431
**PS vs. NC**						
L_CAL		[−18	−54	9]	14.365	6033
L_LING						
R_PCUN						
L_SFG		[−6	33	−3]	11.234	2340
L_IFGorb						
R_PreCG		[33	−27	72]	10.262	1614
R_PoCG						
**Contralesional Thalamus**						
**CS vs. NC**						
Bi_CAL		[−12	−66	9]	12.743	2808
Bi_LING						
L_ITG		[−45	−15	−24]	8.525	175
R_PreCG		[−12	−42	60]	9.718	1723
R_PoCG						
**PS vs. NC**						
Bi_CAL		[−3	33	−3]	12.569	8089
Bi_LING						
R_PoCG		[−30	−18	39]	11.491	2001
R_PreCG						
R_Crus1		[54	−54	−39]	−9.076	148

R, Right; L, Left; CAL, Calcarine; Bi, bilateral; LING, Lingual; MTG, Middle Temporal Gyrus; SMA, Supplementary Motor Area; SMG, SupraMarginal; MOG, Middle occipital gyrus; Crus1, Cerebellum_Crus1; PoCG, Postcentral gyrus; PreCG, Precentral gyrus; SFG, Superior frontal gyrus; PCUN, Precuneus; IFGorb, IFG pars orbitalis; ITG, Inferior temporal gyrus.

### Subfield-group interaction effect on thalamic functional connectivity

**FIGURE 4:**
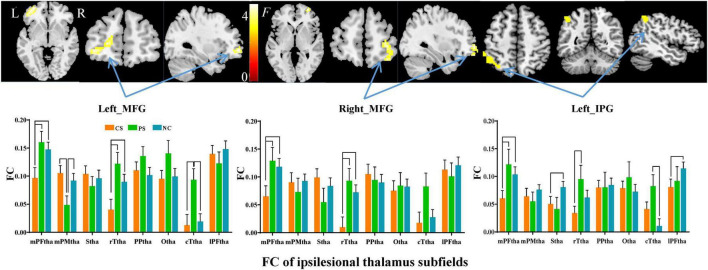
Interaction between groups and subfields on ipsilesional thalamic FC. The brain regions with significant interaction between ipsilesional thalamic subfields and groups (*P* < 0.05, cluster-wise FWE correction). The column chart represents the pos hoc inter-group comparison of subfield-level FC of the ipsilesional thalamus.

**FIGURE 5:**
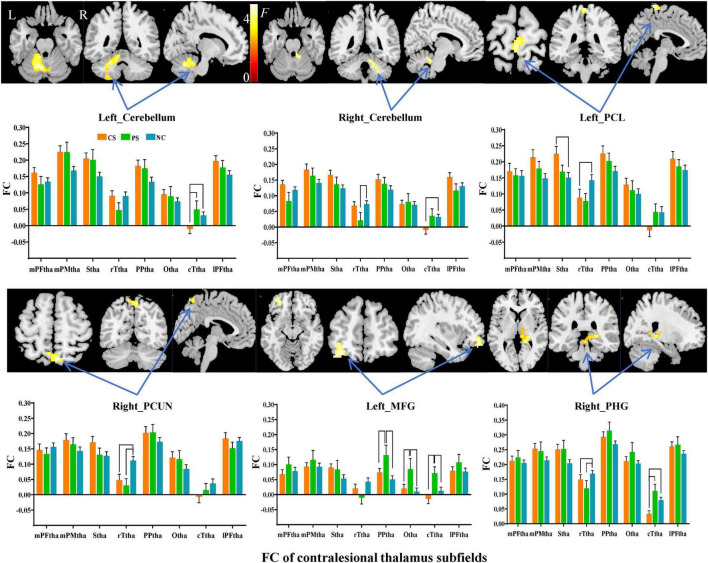
Interaction between groups and subfields on contralesional thalamic FC. The brain regions with significant interaction between contralesional thalamic subfields and groups (*P* < 0.05, cluster-wise FWE correction). The column chart represents the *post-hoc* inter-group comparison of subfield-level FC of the contralesional thalamus.

Subfield-group interaction analyses identified significant subfield-specific functional dysconnectivity after stroke (voxel-wise *P* < 0.001, cluster-wise FWE corrected *P* < 0.05) (Figures 4, 5 and Table 3). Specifically, the ipsilesional thalamic subfields interacted significantly with groups in the bilateral middle frontal gyrus (MFG) and ipsilesional inferior parietal gyrus (IPG) (Figure 4). Further post hoc analyses found that CS patients had significantly lower FC between ipsilesional mPFtha and bilateral MFG/left IPG, as well as between rTtha and contralesional MFG, than the PS and NC. We also found CS patients had significantly lower FC between ipsilesional Stha/IPFtha and ipsilesional IPG than NC, and significantly decreased FC between the ipsilesional rTtha and IPG than the PS. The PS exhibited a notable decrease in FC between the ipsilesional mPMtha and ipsilesional MFG. In contrast, a significant increase in FC was observed between the ipsilesional cTtha and ipsilesional MFG than the CS and NC (*P* < 0.05). For the contralesional thalamus, significant interaction with groups was also found in the ipsilesional CB6, paracentral lobule (PCL), MFG and contralesional CB, precuneus gyrus (PCUN), and parahippocampal gyrus (PHG) (Figure 5). *Post hoc* analyses found that CS patients had significantly lower FC between the contralesional cTtha and bilateral CB/contralesional PHG than the NC. We also found CS patients had significantly lower FC between contralesional rTtha and ipsilesional PCL/contralesional PCUN/contralesional PHG than the NC. The PS showed a significant decrease in FC between contralesional rTtha and contralesional CB/PCUN/PHG. In contrast, a notable increase in FC was shown between the ipsilesional MFG and contralesional PPtha/Otha/cTtha.

**TABLE 3:** Brain regions showing significant thalamic subfield × group interaction on functional connectivity.

Seeds	Targets	Peak MNI coordinate			Peak F	Cluster size (Voxels)
**Contralesional thalamic subfields**		**x**	**y**	**z**		
L_CER6		[−18	−60	−27]	4.510	375
R_ CER8		[−45	27	−39]	3.386	62
L_MFG		[−33	51	−15]	3.514	68
R_PHG		[15	−39	3]	5.030	160
R_PCUN		[6	−66	60]	3.910	62
L_PCL		[−3	−36	75]	3.991	56
**Ipsilesional thalamic subfields**						
R_MFG		[33	60	0]	3.799	57
L_MFG		[−33	54	−3]	3.857	113
L_IPG		[−33	−63	57]	3.904	68

R, Right; L, Left; CER, Cerebelum; MFG, Middle Frontal Gyrus; PHG, ParaHippocampal; IPG, Inferior Parietal Lobule; PCL, Paracentral Lobule; PCUN, Precuneus.

### Correlations between FC changes and clinical variables

We found some correlations between thalamic FC and clinical variables in subcortical stroke patients (controlling for the effects of age, gender, education, and scanners) (*P* < 0.01, uncorrected) (Table 4 and Supplementary Tables 1, 2). For instance, the FC between the ipsilesional thalamus and ipsilesional calcarine was negatively associated with the RAVLT_LR (*r* = −0.371, *P* = 0.003); the FC between the ipsilesional thalamus and ipsilesional Middle cingulate (MCC) showed a negative correlation with the S_ACC (*r* = −0.385, *P* = 0.002); the FC between the ipsilesional thalamus and anterior orbital gyrus (OFCant) showed a negative correlation with the RAVLT_SR and RAVLT_LR (*r* = −0.359, *P* = 0.004; *r* = −0.341, *P* = 0.007); the FC between the ipsilesional thalamus and contralesional superior parietal gyrus (SPG) were negatively associated with the RAVLT_SR (*r* = −0.392, *P* = 0.002); the FC between the ipsilesional thalamus and ipsilesional TPOmid showed a negative correlation with the N_ACC (*r* = −0.357, *P* = 0.005). In the contralesional, the FC between contralesional thalamus and contralesional SFGmedial showed negative correlations with the RAVLT_LR (*r* = −0.335, *P* = 0.008).

**TABLE 4:** The correlations between thalamus FC changes and clinical variables.

	Statistics	FMA	RAVLT_SR	RAVLT_LR	N_ACC	N_RT	S_ACC	S_RT
**Ipsilesional Thalamus**								
L_CAL	r	0.135	−0.240	−0.371	−0.085	0.052	−0.067	0.050
	p	0.301	0.062	**0.003**	0.515	0.692	0.606	0.699
L_MCC	r	−0.043	−0.185	−0.146	−0.313	−0.110	−0.385	−0.107
	p	0.742	0.154	0.260	0.014	0.400	**0.002**	0.412
Bi_OFCant	r	0.055	−0.359	−0.341	−0.133	0.056	−0.174	0.092
	p	0.674	**0.004**	**0.007**	0.305	0.669	0.181	0.481
R_SPG	r	−0.091	−0.392	−0.293	−0.176	0.103	−0.148	0.193
	p	0.486	**0.002**	0.022	0.174	0.429	0.256	0.136
L_TPOmid	r	0.061	−0.207	−0.265	−0.357	0.072	−0.286	−0.051
	p	0.638	0.109	0.039	**0.005**	0.582	0.026	0.696
**Contralesional Thalamus**								
R_SFGmedial	r	0.040	−0.314	−0.335	−0.122	0.042	−0.132	0.073
	p	0.757	0.014	**0.008**	0.347	0.748	0.312	0.575

Black and bold with underline represents *P* < 0.01. R, Right; L, Left; Bi, bilateral; INS, Insula; CAL, Calcarine fissure; MCC, Middle cingulate; OFCant, Anterior orbital gyrus; SPG, Superior parietal gyrus; TPOmid, middle Temporal pole; SFGmedial, Superior frontal gyrus, media.

## Discussion

This study aims to investigate the thalamic subfields’ functional dysconnectivity patterns caused by two types of strokes. We found that the ipsilesional thalamus of capsular stroke patients had abnormally decreased FC with widespread cognitive-related areas while increased FC with sensorimotor areas. In contrast, the ipsilesional thalamus of pontine stroke patients predominantly demonstrated increased FC in these sensorimotor areas and ventral lateral frontal cortex. Even in the contralesional thalamus, we observed similar but less extensive functional dysconnectivity patterns in both the capsular and pontine stroke patients. Finally, we found significant groups × subfields interactions in the bilateral middle frontal gyrus and cerebellum, ipsilesional IPG, PCL and contralesional PCUN, PHG, where capsular vs. pontine stroke demonstrates varied functional dysconnectivity with specific thalamic subfields. These findings suggested that the thalamic functional dysconnectivity after chronic stroke are lesion-location and subfields dependent.

In this study, we found widespread reduced thalamic functional connectivity in both capsular and pontine stroke. Thalamic subfields have dense connections with ipsilesional basal ganglia, ipsilesional cerebral cortex, and even contralesional regions, forming a complex cortico-striato-thalamo-cortical circuit (Kim et al., 2013). Thus, we speculated that lesions within the capsular may directly disrupt the cortico-striato-thalamo-cortical circuit and cause functional disconnectivity (Steiner et al., 2022). Besides, many projection fibers connect the thalamus and the pontine, which relay information between the body and the brain (Chen et al., 2016). Thus, although pontine stroke is far away from the thalamus, it can still impact the pathway of the thalamus and disrupt its functional connectivity. Our findings support the theory that focal brain injury can lead to damage of the functional connectivity of distal regions (Carrera and Tononi, 2014; Lu et al., 2011; Obayashi, 2019). Despite reduced thalamic functional connectivity, both capsular and pontine strokes exhibit an increase in functional connectivity between the bilateral thalamus and the sensory/motor cortices. This is particularly evident in areas such as the postcentral gyrus, precentral gyrus, and calcarine fissure. This pattern may indicate a common compensatory mechanism employed by the brain after a stroke, where increased thalamic connectivity with these sensory/motor regions helps to counteract the resulting functional impairments. This explanation was supported by the research by Zhou et al., who showed that thalamic neurons, when damaged, exhibited reduced firing frequencies but were connected more coherently with some brain regions (Zhou et al., 2014). The areas exhibiting increased functional connectivity with the thalamus may serve as promising targets for personalized rehabilitation strategies in patients suffering from subcortical stroke, providing valuable insights for tailored therapeutic interventions.

It should be noted that the thalamic functional dysconnectivities were heterogeneous between capsular and pontine stroke patients. Specifically, the ipsilesional thalamus of capsular stroke had decreased functional connectivity with widespread frontoparietal cognition-related regions. However, the pontine stroke group did not exhibit reduced FC with. Instead, they showed an increased thalamic FC with the prefrontal cortex, such as OFC and IFG. This finding was in line with previous studies reporting the cortical structural impairment of subcortical stroke patients is highly dependent on the location of infarctions (Guo et al., 2019; Jiang et al., 2017). Although capsular and pons occupy the same projection between the cortex and the brain stem, they engage with the nodal components of the sensory-motor circuit at distinct hierarchical levels (Jiang et al., 2019). Another possibility is that capsular stroke directly impairs the cortical-thalamo-cortical circuits connecting the cognition areas, while pontine stroke does not (Shepherd and Yamawaki, 2021).

We also highlighted that the contralesional thalamus exhibits similar patterns of functional dysconnectivity to the ipsilesional thalamus, albeit to a lesser extent. The thalamus serves as a central hub in a network that communicates between both hemispheres (Dermon, 1994; Yassi et al., 2015). After a stroke, changes in the functional connections between the thalamus and the cortex have been reported in both the ipsilateral and contralateral hemispheres (Hwang et al., 2017; Kim et al., 2013). Moreover, early studies have shown that secondary degeneration of the thalamus following cerebral infarction affected not only the ipsilesional thalamus but also the contralesional thalamus (Yassi et al., 2015). Thus, the contralateral thalamus may play a critical role in facilitating motor recovery after a stroke, underscoring its importance in post-stroke neural rehabilitation (Seitz et al., 1999).

This study further investigated if the thalamic functional dysconnectivities of stroke after stroke are subfield-specific. Our findings highlight significant interactions between stroke groups and thalamic subfields in the bilateral middle frontal gyrus, ipsilesional intraparietal cortex, paracentral lobe, and contralesional precuneus and parahippocampal gyrus. This suggests functional dysconnectivity heterogeneity between thalamic subregions following a stroke. Our research speculates that the locus of lesions within specific thalamic subnetworks may underlie the disrupted connectivity between select thalamic subregions and the cortex (Roy et al., 2022). For example, a marked reduction in connectivity between ipsilesional mPFtha subfields and the bilateral MFG was observed only in capsular strokes, while disconnectivity between ipsilesional mPMtha and MFG was only observed in pontine stroke, resonating with the diaschisis theory which posits functional and physiological changes in distant brain regions post-stroke (Carrera and Tononi, 2014; An et al., 1999). Notably, increased functional connectivity post-stroke is often viewed as a compensatory response, and the strengthened thalamic connectivities were also subfield- and locus-dependent. For instance, in the pontine stroke, enhanced connectivity was shown between contralesional PPtha/Otha and ipsilesional MFG, but was only evident between contralesional Stha and ipsilesional PCL for capsular stroke. These subfield-specific strengthened connections could offer more precise targets for rehabilitation interventions for capsular and pontine stroke patients, respectively.

Finally, we found that the functional dysconnectivity of the thalamus is significantly related to residual cognitive functions in stroke patients, consistent with previous studies (Fama and Sullivan, 2015; Fernandez-Andujar et al., 2014; Jang et al., 2022). For example, the FC between the ipsilesional thalamus and calcarine fissure in stroke patients was negatively associated with long-term verbal memory. As the thalamus-calcarine FC was positive and enhanced in stroke patients, we speculated that this negative FC-cognition association in chronic stroke may suggest a neuroprotection mechanism: chronic stroke patients need higher thalamic-calcarine FC to compensate for more severe verbal memory deficits, although this “compensation” is not complete. We also identified a weak positive correlation between thalamic-SMG FC and numeric working memory in stroke patients, while the thalamic-SMG FC was decreased in stroke, meaning that worse damage in thalamus-SMG FC corresponds to a more severe working memory deficit. Previous studies have shown lesions in the frontoparietal network were associated with working memory deficits (Lugtmeijer et al., 2021). The SMG is a central part of the frontoparietal network and is associated with working memory performance (Cruz-Gomez et al., 2014). In summary, this study suggest that the thalamic-cortial circuits participant multi-demensional higher-tier cognition task may contribute to the rehabilitation potential of these cognitive impairments.

There exist some limitations within the current study. Firstly, the number of patients with pontine stroke included in the sample is comparatively limited. After strict screening of the inclusion and exclusion criteria, only 20 patients with ipsilesional pontine stroke were carefully selected for inclusion. In future work, the data of patients with pontine stroke need to be supplemented. Secondly, the present study is a cross-sectional experimental design, which cannot reveal the dynamic evolution of thalamic functional connectivity and its correlation with cognitive changes. Longitudinal data from a large sample may provide more useful information to reveal the correlation between changes in thalamic functional connectivity and cognitive function after stroke. Additionally, our research data originate from three hospitals with their respective different MRI scanners. Although we have considered the different MRI scanners as nuisance covariates during statistics, it is still difficult to completely eliminate the bias caused by site heterogeneities. In the further, we will expand the sample size and use a unified MRI scanner for data collection to further validate our findings. Finally, in this study, we recruited only patients with infarction lesions in the capsular and pons regions of the left hemisphere to reduce the effects of lesion heterogeneity. We acknowledge that this approach is a “double-edged sword” that may limit the generalization of our findings. However, it should be noted that lesion heterogeneity in stroke patients is difficult to avoid in clinical studies, and our strategy is commonly adopted by previous stroke studies (Fan et al., 2013; Hope et al., 2017; Kalenine et al., 2010).

In summary, our findings suggest that thalamic functional dysconnectivity patterns after chronic stroke are lesion-location and subfields dependent. Moreover, functional dysconnectivity was shown in both the ipsilesional and contralesional thalamus with similar patterns.

## Data Availability

The original contributions presented in the study are included in the article/Supplementary material, further inquiries can be directed to the corresponding author.

## Footnotes

1
http://rfmri.org/DPABI

2
https://www.fil.ion.ucl.ac.uk/spm/software/spm12/

3
https://atlas.brainnetome.org/
