# Supplementary material for: Thalamic functional dysconnectivity in patients with left-hemisphere chronic capsular and pontine stroke
Source: Front Neurosci. 2024 Oct 15;18:1451307. doi: 10.3389/fnins.2024.1451307 (PMC11519739; doi:10.3389/fnins.2024.1451307)
Supplement: Supplementary file 1 [file Data_Sheet_1.PDF]

Table 1. The correlations between ipsilesional thalamus FC changes and clinical variables.

Brain regions	statistics	FMA	RAVLT_SR	RAVLT_LR	N_ACC	N_RT	S_ACC	S_RT
L_CAL	r	0.135	-0.240	-0.371	-0.085	0.052	-0.067	0.050
	p	0.301	0.062	**0.003**	0.515	0.692	0.606	0.699
R_CAL	r	0.024	-0.118	-0.183	-0.198	-0.005	-0.051	-0.058
	p	0.857	0.366	0.158	0.126	0.969	0.694	0.655
L_CER8	r	0.146	-0.316	-0.329	0.095	0.038	-0.151	0.085
	p	0.261	**0.013**	**0.010**	0.464	0.770	0.247	0.515
L_CERCRU2	r	-0.036	-0.260	-0.305	0.091	0.127	-0.013	0.144
	p	0.784	**0.043**	**0.017**	0.487	0.329	0.923	0.270
L_MCC	r	-0.043	-0.185	-0.146	-0.313	-0.110	-0.385	-0.107
	p	0.742	0.154	0.260	**0.014**	0.400	**0.002**	0.412
L_IFGoperc	r	-0.093	-0.206	-0.234	-0.103	0.074	-0.201	0.038
	p	0.476	0.112	0.070	0.429	0.573	0.120	0.774
PFCventmed	r	0.107	-0.309	-0.237	-0.096	-0.014	-0.273	0.014
	p	0.410	**0.015**	0.066	0.461	0.915	**0.033**	0.913
L_MFG	r	0.055	-0.172	-0.198	-0.075	0.065	-0.068	0.030

	p	0.676	0.184	0.127	0.566	0.619	0.602	0.817
L_SFG	r	-0.009	-0.083	-0.101	-0.132	0.083	-0.153	-0.114
	p	0.944	0.526	0.440	0.312	0.526	0.240	0.380
R_HIP	r	0.064	0.197	0.112	0.039	0.040	0.080	0.133
	p	0.625	0.127	0.390	0.765	0.758	0.541	0.307
L_INS	r	-0.110	-0.208	-0.253	-0.091	0.090	-0.185	0.047
	p	0.400	0.108	**0.049**	0.484	0.492	0.154	0.717
R_INS	r	-0.119	-0.170	-0.216	0.024	0.078	-0.143	0.111
	p	0.362	0.190	0.094	0.856	0.549	0.271	0.396
L_LING	r	0.057	0.076	-0.094	0.011	0.170	0.180	0.125
	p	0.661	0.562	0.471	0.933	0.190	0.165	0.336
L_MOG	r	0.036	-0.157	-0.174	-0.052	0.155	-0.083	0.115
	p	0.782	0.227	0.179	0.692	0.232	0.526	0.376
R_MOG	r	0.139	0.074	-0.013	-0.085	0.025	-0.101	0.023
	p	0.285	0.570	0.920	0.514	0.847	0.437	0.860
OFCant	r	0.055	-0.359	-0.341	-0.133	0.056	-0.174	0.092
	p	0.674	**0.004**	**0.007**	0.305	0.669	0.181	0.481
R_PCL	r	0.065	-0.204	-0.285	-0.100	0.038	-0.233	0.086

	p	0.620	0.115	**0.026**	0.443	0.771	0.071	0.509
L_SPG	r	0.010	-0.227	-0.164	-0.064	0.042	0.030	0.051
	p	0.937	0.078	0.208	0.625	0.747	0.820	0.694
R_SPG	r	-0.091	-0.392	-0.293	-0.176	0.103	-0.148	0.193
	p	0.486	**0.002**	**0.022**	0.174	0.429	0.256	0.136
L_PoCG	r	0.254	-0.141	-0.194	-0.132	-0.103	-0.277	-0.116
	p	0.05	0.278	0.134	0.310	0.431	**0.031**	0.373
L_PreCG	r	0.106	-0.252	-0.264	-0.213	0.005	-0.279	-0.019
	p	0.415	0.050	**0.040**	0.099	0.967	**0.029**	0.886
R_PreCG	r	0.107	-0.218	-0.197	-0.061	0.047	-0.241	0.068
	p	0.412	0.091	0.128	0.638	0.717	0.061	0.604
L_SMG	r	-0.028	-0.137	-0.283	0.261	0.092	0.028	-0.041
	p	0.829	0.291	**0.027**	**0.042**	0.482	0.833	0.754
R_SMG	r	-0.086	-0.240	-0.327	0.224	0.165	0.034	0.161
	p	0.511	0.063	**0.010**	0.082	0.204	0.795	0.215
L_TPOmid	r	0.061	-0.207	-0.265	-0.357	0.072	-0.286	-0.051
	p	0.638	0.109	**0.039**	**0.005**	0.582	**0.026**	0.696
R_TPOmid	r	0.127	-0.152	-0.102	-0.146	-0.084	-0.294	-0.143

	p	0.329	0.242	0.435	0.262	0.519	**0.021**	0.270

Note: Black and bold represents $P < 0.05$, with underline represents $P < 0.01$. Abbreviations:INS=Insula; CAL=Calcarine;SMG=SupraMarginal gyrus; MOG=Middle occipital gyrus; MCC= Middle cingulate; OFCant=Anterior orbital gyrus; SPG= Superior parietal gyrus; TPOmid= middle Temporal pole; PoCG=Postcentral gyrus; PreCG=Precentral gyrus; HIP=Hippocampus; LING=Lingual; IFGoperc=Inferior frontal gyrus, opercular part; PFCventmed=Superior frontal gyrus, medial orbital; SFG=Superior frontal gyrus; MFG=Middle frontal gyrus; PCL=Paracentral Lobule; CERCRU2=Crus II of cerebellar hemisphere; CER8=Lobule VIII of cerebellar hemisphere

Table 2. The correlations between contralesional thalamus FC changes and clinical variables

Brain regions	statistics	FMA	RAVLT_SR	RAVLT_LR	N_ACC	N_RT	S_ACC	S_RT
CAL	r	0.072	-0.101	-0.244	-0.168	-0.032	-0.163	-0.029
	p	0.582	0.437	0.059	0.195	0.806	0.208	0.827
L_CAL	r	0.155	-0.124	-0.273	-0.124	-0.071	-0.160	-0.043
	p	0.231	0.340	**0.033**	0.340	0.589	0.219	0.745
R_CRUS1	r	-0.056	0.043	0.098	0.046	0.014	0.148	0.041
	p	0.667	0.740	0.450	0.724	0.913	0.256	0.754
L_PFCventmed	r	0.099	-0.204	-0.227	-0.107	-0.034	-0.157	0.015
	p	0.449	0.115	0.078	0.410	0.794	0.226	0.911
L_MFG	r	0.071	-0.198	-0.297	-0.152	-0.013	-0.065	-0.018
	p	0.584	0.126	**0.020**	0.242	0.918	0.620	0.891
R_MFG	r	0.025	-0.087	-0.108	0.200	-0.033	-0.049	0.061
	p	0.849	0.504	0.409	0.123	0.801	0.710	0.639
L_SFG	r	-0.114	-0.189	-0.104	-0.132	0.188	-0.091	0.007
	p	0.380	0.144	0.424	0.310	0.146	0.484	0.958
R_SFGmedial	r	0.040	-0.314	-0.335	-0.122	0.042	-0.132	0.073

	p	0.757	**0.014**	**<u>0.008</u>**	0.347	0.748	0.312	0.575
L_MOG	r	0.045	-0.001	-0.038	-0.053	-0.029	-0.127	0.066
	p	0.729	0.992	0.773	0.688	0.825	0.329	0.614
L_SPG	r	0.006	-0.110	-0.048	-0.106	0.034	0.069	0.052
	p	0.963	0.399	0.715	0.415	0.798	0.595	0.688
L_PoCG	r	0.119	-0.200	-0.294	-0.280	-0.001	-0.270	0.003
	p	0.362	0.123	**0.022**	**0.029**	0.993	**0.035**	0.979
R_PreCG	r	0.168	-0.194	-0.285	-0.211	-0.037	-0.310	-0.006
	p	0.194	0.135	**0.026**	0.102	0.776	**0.015**	0.963
L_PCUN	r	0.177	-0.086	-0.188	-0.226	-0.102	-0.229	-0.078
	p	0.171	0.512	0.148	0.079	0.433	0.076	0.552
L_REC	r	0.177	-0.148	-0.240	-0.224	-0.087	-0.295	-0.060
	p	0.172	0.253	0.062	0.082	0.507	**0.021**	0.645

Note: Black and bold represents $P < 0.05$, with underline represents $P < 0.01$. Abbreviations: CAL=Calcarine; MOG=Middle occipital gyrus; SFGmedial=Superior frontal gyrus, medial; PCUN=Precuneus; REC=Gyrus rectus; CRUS1=Crus I of cerebellar hemisphere

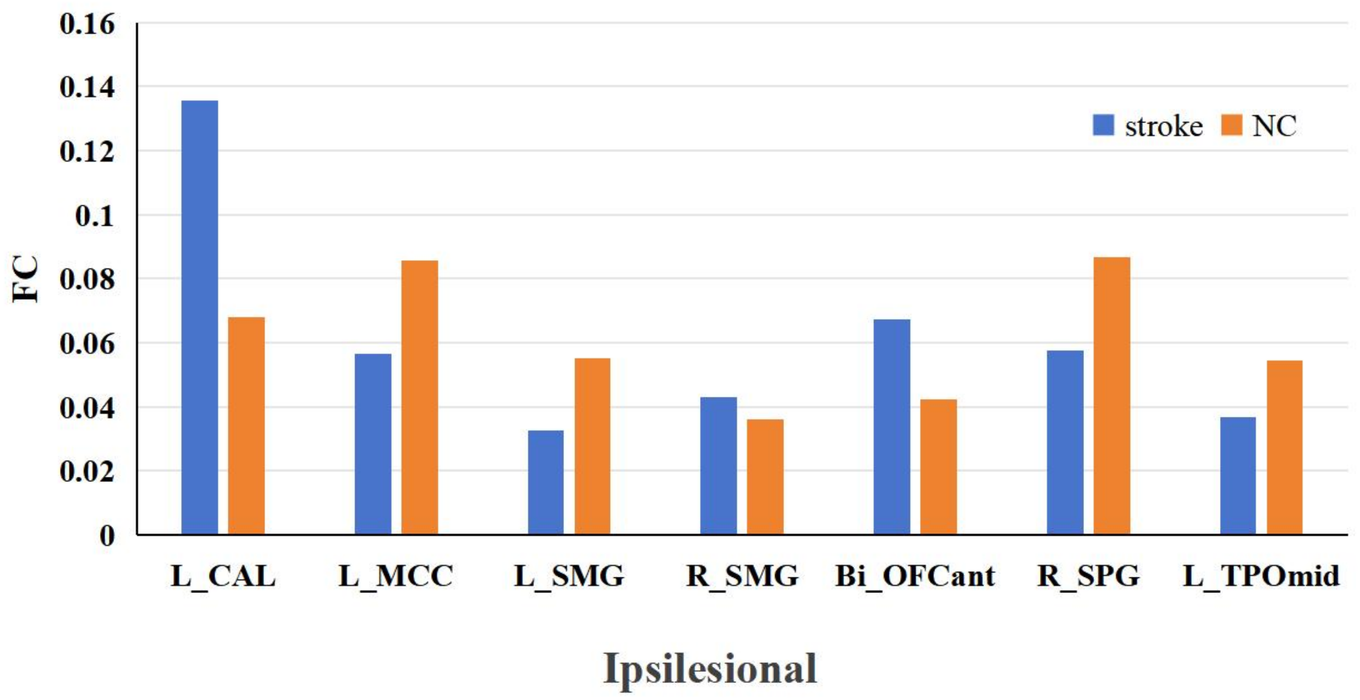

Supplementary Figure 1: The mean functional connectivity across ipsilesional thalamic subfields of stroke and normal controls.

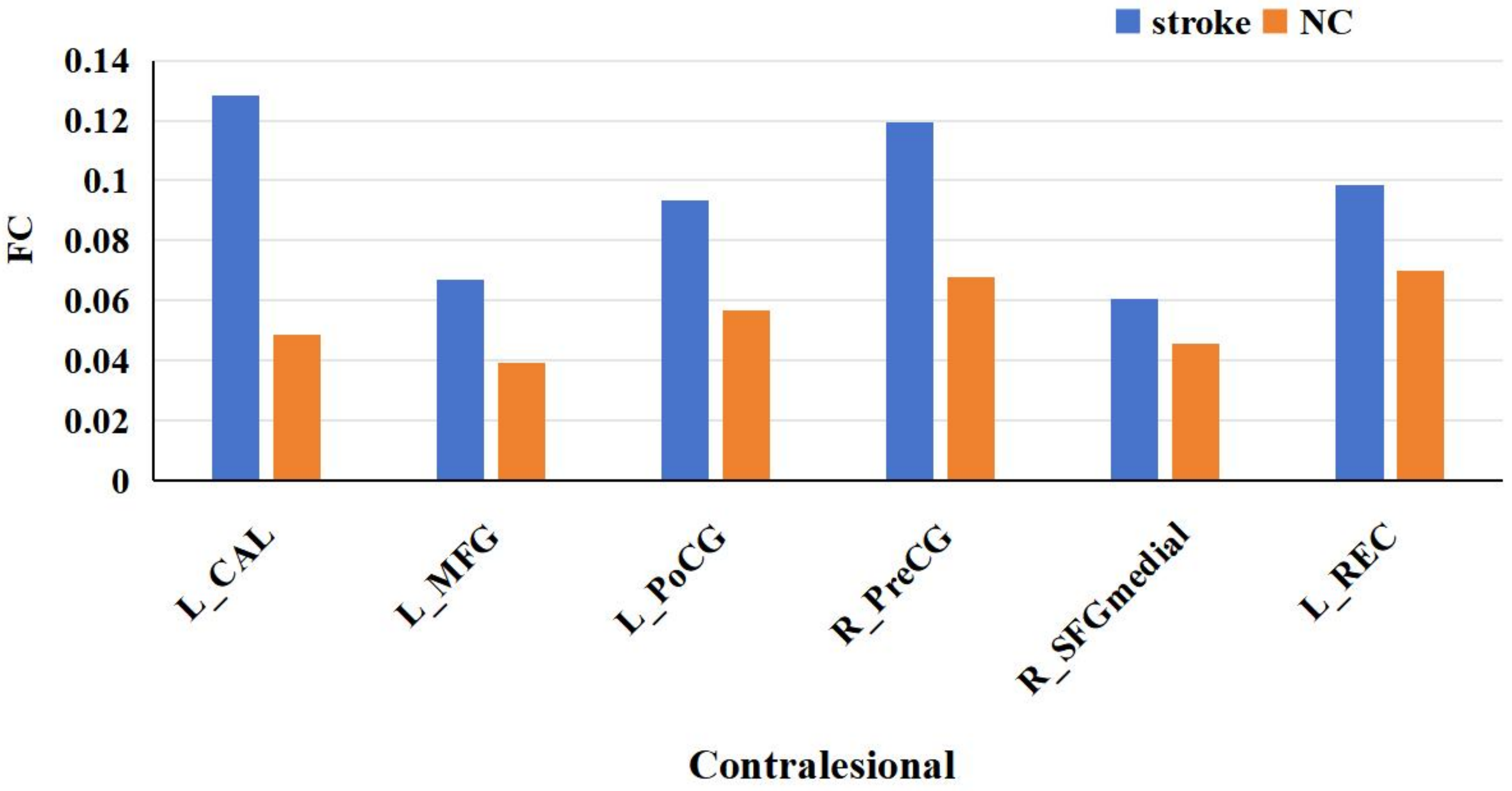

Supplementary Figure 2: The mean functional connectivity across contralesional thalamic subfields of stroke and normal controls.
